# Northern wild-rice (manoomin) is limited by a warm lake and the co-occurrence of native and non-native aquatic plants in Northern Michigan, USA

**DOI:** 10.3389/fpls.2026.1830267

**Published:** 2026-05-01

**Authors:** Shane C. Lishawa, Megan M. Wenner, Danielle L. Fegan, Alexandra J. Risdal, Alison K. Varty, Brian M. Ohsowski

**Affiliations:** 1 School of Environmental Sustainability, Loyola University Chicago, Chicago, IL, United States; 2 Natural Resources Division, Sault Ste. Marie Tribe of Chippewa Indians, Sault Ste. Marie, MI, United States; 3 College of the Siskiyous, Weed, CA, United States

**Keywords:** ecological restoration, *Hydrocharis morsus-ranae*, plant competition, *Pontederia cordata*, *Typha*, *Zizania palustris*

## Abstract

**Introduction:**

*Zizania palustris* (northern wild-rice, manoomin), is foundational to the cultural heritage of indigenous peoples in the Great Lakes region of North America, provides habitat for diverse fauna, and its regional distribution and abundance have been substantially reduced. Restoration and sustainable stewardship of manoomin is a shared goal of tribes across the region.

**Methods:**

Our research objective was to refine our understanding of manoomin’s ecological niche and competitive dynamics in order to improve restoration outcomes through four related studies: Study one (Q1) evaluated differences between lakes with differing seeding successes; study two (Q2), quantified manoomin growth and environmental conditions within mixed aquatic plant communities; study three (Q3) tested experimental manoomin seeding within three dominant aquatic plant types; and study four (Q4) evaluated the indirect effects of shading and invasive plant (*Typha × glauca*) litter on manoomin.

**Results:**

We found that the warmer of two lakes sampled had dramatically lower manoomin productivity (Q1); *Pontederia cordata* was negatively associated with manoomin (Q2); manoomin was more abundant and larger in *Nuphar variegata* stands (Q3); and litter from *T. × glauca* and a proxy for floating plant cover reduced germination and early growth (Q4).

**Discussion:**

Our findings illustrate that both native and invasive plants can be detrimental to the germination, growth, and productivity of manoomin. *T. × glauca* and its litter caused particularly strong negative effects, indicating that invasive plant management is necessary for sustainable restoration. Our study points toward areas for future manoomin research and highlights the multifaceted threats of global change factors (e.g., climate change and non-native macrophytes) on plant species of high cultural importance.

## 1 Introduction

*Zizania palustris* (northern wild-rice, Manoomin/Mnomen in Anishinaabemowin), an annual emergent grass, is one of three North American wild-rice species (Biesboer, 2019). Manoomin is foundational to the cultural heritage of indigenous peoples in the Great Lakes region of North America, and continues to hold great cultural and economic significance (Barton, 2018). Manoomin also serves as a food and habitat resource for many species of wetland birds, fish, amphibians, and invertebrates. Prior to European colonization, manoomin’s historic range extended throughout the Great Lakes region of Canada and the upper Midwest of the United States for 6,000 or more years (Boyd et al., 2013). The present extent of manoomin has been substantially reduced due to wetland destruction, changes in hydrology and water quality, climate change, and invasive plant encroachment (Pillsbury and McGuire, 2009; Biesboer, 2019; Dysievick, 2019; McGilp et al., 2023). A recent analysis of off-reservation manoomin harvesting data between 1985–2020 by Nyblade et al. (2025) identified a decline in available manoomin for tribal harvest by 5-7% annually over this period; hydrological changes driven by climate change are largely responsible for these reductions. The loss of manoomin has been particularly acute in Michigan where only an estimated 1% of historically documented beds still exist today (Barton, 2018). Restoration and sustainable stewardship of manoomin is a shared goal of many tribes across the Great Lakes region.

Manoomin thrives in slow-flowing rivers and shallow lakes with organic sediments and water levels generally between 10–100 cm (Lee, 2002; Biesboer, 2019). Its seeds require a cold and wet stratification period to break the seed coat and allow germination (Atkins, 1986). Overwintering occurs in benthic sediments of lakes and rivers, where temperatures remain around 4 °C for at least three to five months (Cardwell et al., 1978; McGilp et al., 2023). Manoomin populations are sensitive to changes in water quality, water temperature, pH, salinity, pollutants and hydrological shifts (McGilp et al., 2023). For example, a pH greater than 8.5 and water temperatures greater than 23 °C may be detrimental to seed germination and growth (Varty et al., 2024). Manoomin is also susceptible to major and rapid changes in water level during the growing season (Biesboer, 2019; Dysievick, 2019). Rapid increases in water depth can uproot fragile seedlings during the floating leaf stage (Raster and Hill, 2017), while rapid decreases in water depth can cause immature plant mortality due to loss of water buoyancy (Vennum, 1988). Furthermore, when water levels are too high, sufficient sunlight cannot penetrate to the seeds and seedlings may not be able to reach the surface (Barton, 2018; VanZomeren et al., 2023). When water levels are too low, competing emergent species, including invasives, can more readily establish (VanZomeren et al., 2023). Many questions remain about how such differences in environmental conditions across lakes impact the long-term stability of manoomin populations.

Manoomin does not often occur in mixed-species stands, and is not generally considered a good competitor with established species of perennial emergent wetland plants (Atkins, 1986; Lee, 1986). Native competitors include species such as *Pontederia cordata* (pickerel-weed), *Eleocharis palustris* (spike-rush), *Sparganium eurycarpum* (common bur-reed), *Alisma triviale* (northern water-plantain), *Nuphar variegata* (yellow pond-lily), *Nymphaea odorata* (sweet-scented water lily) and *Schoenoplectus acutus* (hardstem bulrush) (Ransom and Oelke, 1982; Lee, 1986; Clay and Oelke, 1987). Interspecific competition for resources reduces manoomin germination, growth, survival rate, and seed yield (Ransom and Oelke, 1982; Clay and Oelke, 1987). Mechanisms by which competing plants impede manoomin growth include differential nutrient uptake rates, competition for space or sunlight, and greater tolerance of water fluctuations (Clay and Oelke, 1987; Barton, 2018; Dysievick, 2019).

Competition with dominant invasive plants may be a greater threat to manoomin populations than competition with native species. *Typha* × *glauca*, *T. angustifolia*, and their advanced generation hybrids (hereafter *Typha*) are dominant invasive plants that form dense monotypic stands, they can survive in a variety of water depths, and exhibit a fast rate of production of aboveground tissue (Bansal et al., 2019). Standing *Typha* litter is slow to decompose and, over time, a high volume of partially decomposed *Typha* litter accumulates in invaded wetlands. Accumulated litter decreases light penetration and the germination and growth of native plants (Mitchell et al., 2011; Larkin et al., 2012a). Accumulating *Typha* litter also alters sediment chemical and physical characteristics (Larkin et al., 2012a; Dysievick, 2019). Another aquatic invasive plant of potential concern for manoomin populations is *Hydrocharis morsus-ranae* (European frog’s-bit; EFB). EFB is a floating macrophyte, native to Europe, that is presently spreading throughout the Great Lakes region (Catling et al., 2003; Zhu et al., 2018). It grows in dense, interwoven floating mats that can decrease the diversity of native macrophytes, particularly by reducing available light below the mat (Catling et al., 1988, Catling et al., 2003). EFB presence can also limit nutrient and dissolved oxygen availability and reduce water flow (Zhu et al., 2018). Furthermore, wetland invasion by EFB is facilitated by the prior establishment of *Typha*, which creates suitable EFB habitat by sheltering against wind and waves (Monks et al., 2019). While it is widely accepted that *Typha* is a threat to manoomin, the direct effects of *Typha* and its litter on manoomin germination and early growth have not been studied in detail.

This manuscript seeks to refine our understanding of manoomin habitat niche and competitive dynamics to improve restoration outcomes in northern Michigan, USA by integrating four independent studies. Specifically, we sought to answer the following questions: What are the differences in environmental conditions between two geographically proximal northern Michigan lakes: French Farm Lake (which harbors robust manoomin), and Wycamp Lake (which has not been able to sustain a thriving population), and how do these differences lead to dissimilar outcomes for their respective populations of manoomin (*Q1: Lake-to-lake comparison)*? What effect do existing stands of common wetland plants have on the distribution and abundance of manoomin in a northern Michigan lake (French Farm Lake) with a self-sustaining population (*Q2: dominant plant type observational study)*? How does manoomin germinate and persist when seeded among different dominant native plant types *(Q3: dominant plant type experimental seeding study)*? What effects do the presence of a common wetland invasive plant (*Typha* × *glauca* via its litter) and the shading associated with floating plant cover have on manoomin germination and early growth *(Q4: litter and cover mesocosm experiment)*? We hypothesized that: 1) the environmental conditions that are most suitable for the germination and growth of manoomin (e.g., circumneutral pH, cool temperatures) will differ between Wycamp Lake and French Farm Lake; 2) measures of manoomin density and productivity will vary based on the dominant plant species with which manoomin co-occurs; 3) seeding manoomin within existing native plant stands will result in variable success as dense floating plants likely result in the lowest density and productivity in the following season; 4) manoomin germination and growth rates will be negatively impacted by the presence of invasive *Typha* litter and shading.

## 2 Methods

### 2.1 Study sites

We conducted field studies (Q1, Q2, Q3) at two sites, French Farm Lake and Wycamp Lake located in northern Michigan, USA. The Little Traverse Bay Bands of Odawa Indians (LTBB) established a manoomin population at French Farm Lake through repeated seeding at 56.1 kg/ha (50 lb/acre) in areas of suitable water depth (Varty et al., 2024). Manoomin is now widespread and self-sustaining in the absence of active seeding. Similar seeding methods were used by LTBB in Wycamp Lake over the same period, but manoomin growth was patchy and sparse throughout the lake. Manoomin that did grow at Wycamp Lake was generally spindly compared to the healthy plants at French Farm Lake (Varty et al., 2024).

### 2.2 (Q1) lake-to-lake comparison

We conducted an observational field study in 2023 quantifying differences in environmental conditions and manoomin growth between French Farm Lake and Wycamp Lake to gain insight into varied manoomin growth responses. Existing stands of manoomin were identified in both lakes and we selected a subset of stands for our study. We established 10 m × 10 m plots within each stand (French Farm Lake: *n* = 6; Wycamp Lake: *n* = 5) and randomly located three 1 m^2^ subplots within each plot. On July 25, 2023, we recorded the number of manoomin culms, percent cover, height of a randomly selected culm, and a suite of environmental variables in each subplot. We used a 3 m graduated PVC pole to measure sediment depth and water depth to the sediment surface. We measured water temperature, pH, specific conductivity, dissolved oxygen, and oxidation-reduction potential with a YSI Professional Plus Multiparameter Instrument (Yellow Springs Instruments, Yellow Springs, Ohio). We randomly collected one manoomin specimen per subplot, measured its stem height, separated its roots and shoots, dried the tissue to a constant weight at 60 °C, and weighed all samples. We used the resulting biomass and height data to calculate shoot and root biomass, root: shoot ratio, and spindliness (height: weight) for each plant.

### 2.3 (Q2) dominant plant type observational study

We conducted an observational field study at French Farm Lake in late July 2021 – 2022, to assess manoomin abundance and growth within different dominant plant types. We established plots in locations that contained manoomin and were dominated by one of four plant types: manoomin (*n* = 11), *Pontederia cordata* (pickerel-weed; *n* = 10), *Schoenoplectus acutus* (hardstem bulrush; *n* = 10), or water lilies (i.e., *Nuphar variegata* or *Nymphaea odorata*; *n* = 3). We sought to standardize water depths and sediment conditions across plots by selecting locations with similar water depths (0.25 – 1.0 m) and similar organic substrate depths (> 1 m). In each plot, we collected data from three 1 m^2^ subplots. We recorded vegetation composition data (aerial cover of all species), quantified manoomin stem density (stems/m^2^) and cover (aerial cover %), and collected a suite of environmental variables (water temperature [°C], water depth [cm], pH, oxidation-reduction potential [ORP; mV]) in all subplots. We calculated plant diversity metrics (Shannon diversity [H’] and species richness) from plant composition data.

### 2.4 (Q3) dominant plant type experimental seeding study

We conducted a field experiment in French Farm Lake in 2023 and 2024 to explore the germination and growth dynamics of manoomin seeded in the presence of native plant species. We identified stands of different existing dominant plant types and open water areas without manoomin present: open water, *Nuphar variegata*, *Pontederia cordata*, and *Schoenoplectus acutus*. We sought to standardize water depths and sediment conditions across plant communities by selecting locations with similar water depths (0.25 – 1.0 m) and similar organic substrate depths (> 1 m). We established five 5 m × 5 m plots per community type. Within each plot, we broadcast spread 0.23 kg of manoomin seeds in mid-August 2023. The rate of seeding slightly exceeded the typical recommended rate of 56.1 kg/ha (50 lb/acre) (David, 2018). In August 2024, we collected vegetation composition, manoomin stem density and cover data from three 1 m^2^ subplots per plot, and derived plant diversity metrics from vegetation cover data, as described above.

### 2.5 (Q4) litter and shading mesocosm experiment

We conducted a mesocosm study at the University of Michigan Biological Station during summer of 2023 to test the effect of three cover treatments (*Typha* litter; low cover; high cover) on manoomin germination and early growth. We created mesocosms following the design in Varty et al. (2024) using 11.4 L buckets that were 30.0 cm tall with 21.0 cm bottom diameter. Within each mesocosm we added 10 cm of organic sediment collected from French Farm Lake and uniformly sieved to remove plant matter and seeds. We filled each mesocosm to 25 cm depth with ground water and maintained water levels daily throughout the experiment. We applied one of four treatment levels to each mesocosm: control, low cover (40% cover), high cover (80% cover), and *Typha* litter (*Typha* litter addition). *Typha* litter addition treatments involved adding a typical depth of dried *Typha* × *glauca* litter (approximately 10 cm; 1.9 L) collected from a *Typha* stand in Cheboygan Marsh (Cheboygan, Michigan, USA), cut into 10 cm lengths, and applied to the surface to each treatment mesocosm. Low cover and high cover treatment levels simulated two levels of cover, meant to reflect a sparse (40%) and well-established (80%) floating plant population. We used blue, 2.54 cm thick, rigid insulation board (Dow UtilityFit Styrofoam Insulation; Dow Chemical, Midland MI, USA) cut into 5 cm diameter circles as a proxy for floating plant cover in order to standardize and isolate effect of shading on manoomin. We applied the cut foam board to the surface of buckets to cover 40% or 80% of the water’s surface area. We established seven replicate experimental blocks, containing a mesocosm of each treatment (7 blocks × 4 mesocosms). We submerged all mesocosms to 25 cm depth (5 cm below the mesocosm rim) within 7 (one per-block) 100 L plastic tanks (86.0 cm length × 47.6 cm width × 33.0 cm height). We sunk the plastic tanks into the ground to provide thermal insulation. We maintained cool, stable temperatures (~ 20 °C) by continually running ground water through each tank. We introduced 30 manoomin seeds into each of the 28 mesocosms. The growing period lasted from July 24, 2023 to August 7, 2023. We quantified manoomin germination and survival, along with water chemistry in the mesocosms, weekly, and maintained water levels daily. At the conclusion of the experiment, we measured root and shoot lengths, and root counts for each seedling. We dried all samples to a constant weight at 60 °C to estimate shoot and root biomass for each seedling.

### 2.6 Statistical analyses

We used linear mixed-effects (LME) models to evaluate differences in a suite of plant and environmental variables between lakes with subplot within plot ID as a random factor (Q1), to assess manoomin abundance and growth within different wetland plant community types with year as a random factor (Q2), to explore the germination and growth dynamics of manoomin seeded in the presence of various native plant species with subplot within plot as a random factor (Q3), and to test the effects of *Typha* litter and shading with treatment block as a random factor (Q4). In each LME, we estimated model explained variance by calculating marginal (fixed effects only) and conditional (fixed effect and random effects) R^2^ values. We log or square root transformed response variable data to meet LME model assumptions of residual normality and homoscedasticity, when necessary. We assessed differences among treatments in all experiments using the estimated marginal means approach and Tukey’s honestly significant differences *post hoc* tests. Additionally, we evaluated the co-occurrence of plant species (Q2) using probabilistic species co-occurrence analysis (Veech, 2013) and visually evaluated unseeded plant community composition using nonmetric multidimensional scaling (NMDS) with Bray-Curtis dissimilarity calculations. We used vector analysis to test the relationships between plant communities and environmental factors by fitting linear trends on the NMDS ordination using random permutations of the data (*n* = 1000). We conducted all statistical analysis in R 4.1.1 (R Core Team, 2021), using the *lme4* package for LME models (Bates, 2016), the *emmeans* package for calculating estimated marginal means (Lenth et al., 2023), the *vegan* package for plant diversity calculations and NMDS analyses (Oksanen et al., 2020), and the *cooccur* package for species co-occurrence (Griffith et al., 2016). Significance for all tests was α < 0.05.

## 3 Results

### 3.1 (Q1) lake-to-lake comparison

**Figure 1:**
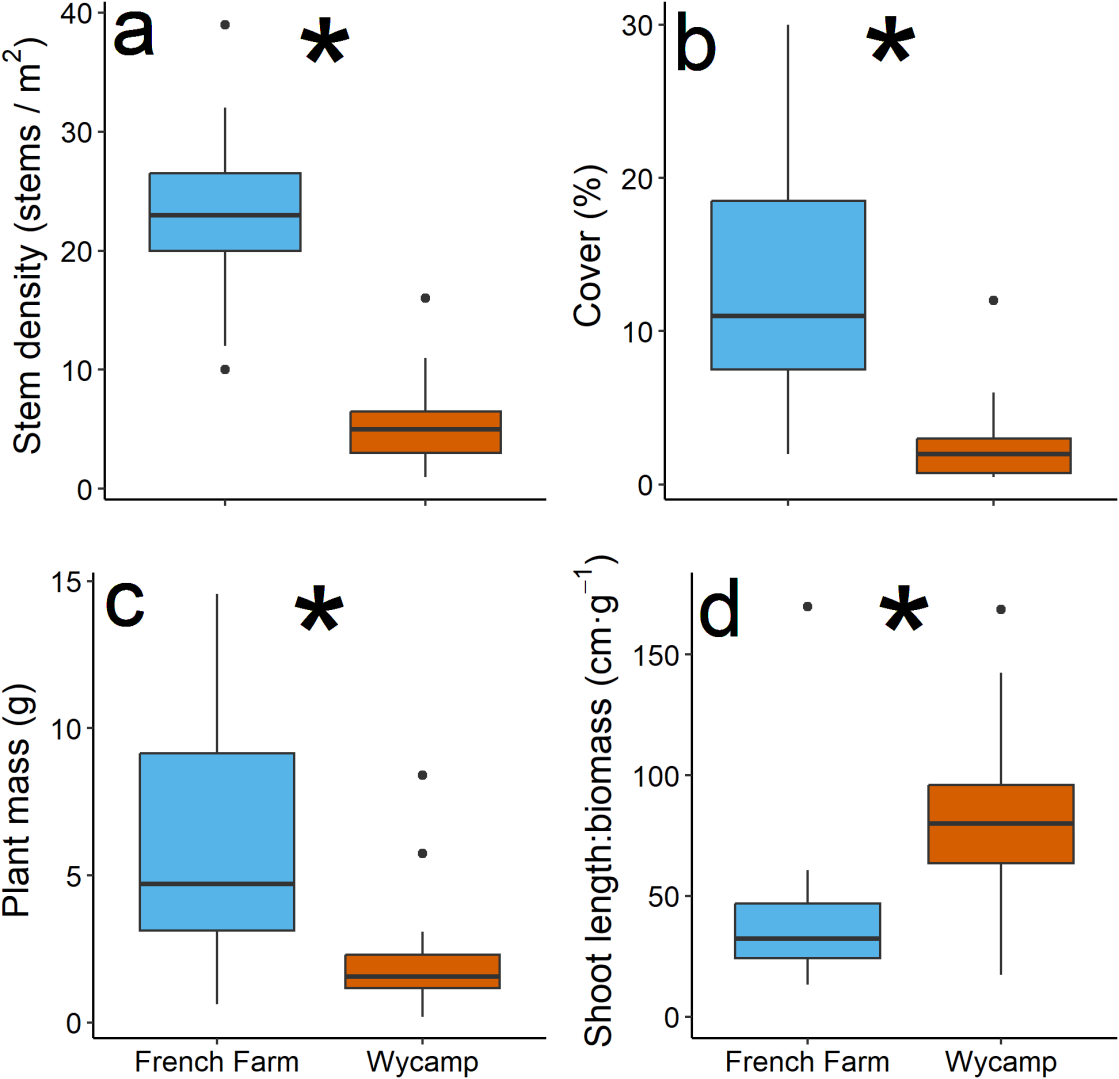
Response variables in French Farm Lake and Wycamp Lake, based on manoomin stem density **(a)**, percent coverage **(b)**, plant mass **(c)**, and spindliness (stem height: stem mass) **(d)**. Asterisk indicates statistically significant differences between lakes.

Nearly all measures of manoomin differed between lakes: French Farm mean stem density and cover were over 4-times greater; mean stem height was more than 35% greater; mean stem mass was over 3-times greater; and mean root mass was more than double Wycamp manoomin (**Figure 1**). Further, Wycamp manoomin plants had significantly greater spindliness (stem height: stem mass) than French Farm individuals. Of the measured environmental variables, water temperature was higher in Wycamp Lake, Cl^-^ was greater in French Farm Lake, and ORP trended higher in French Farm Lake (*p* = 0.07; **Table 1**). Water depth, pH, and NH_4_^+^ did not differ among lakes (all *p* > 0.05; **Table 1**), and sediment organic depth was greater than 1 m at all plots.

**Table 1:** Response variables (estimated marginal mean ± SE) within French Farm and Wycamp Lakes (northern Michigan, USA).

Variable	Model fit (R^2^)		Lake (estimated marginal mean ± SE)		
Manoomin metrics	Marginal	Conditional	French farm (*n* = 15)	Wycamp (*n* = 15)	*p* – value
Stems/m^2^	0.68	0.77	23.2 ± 1.95	5.6 ± 1.95	< 0.001
Percent cover	0.69	0.85	13.3 ± 1.69	2.7 ± 1.69	< 0.01
Height (cm)	0.64	0.83	146 ± 7.14	109 ± 7.14	< 0.01
Plant weight total (g)	0.26	0.56	6.36 ± 1.22	2.23 ± 1.22	0.04
Shoot mass (g)	0.27	0.58	5.3 ± 0.89	1.8 ± 0.50	0.04
Root mass (g)	0.17	0.40	1.10 ± 0.25	0.41 ± 0.25	0.09
Root: shoot	0.00	0.00	0.17 ± 0.03	0.17 ± 0.03	0.94
Spindliness (stem height: stem mass)	0.28	0.51	34.9 ± 8.54	87.3 ± 21.37	0.03
Environmental metrics					
Water temperature (°C)	0.54	0.81	26.2 ± 0.31	27.9 ± 0.31	< 0.01
Water depth (cm)	> 0.01	0.86	73.5 ± 5.88	72.3 ± 4.30	0.93
Specific conductivity (µS/cm)	0.07	0.23	288 ± 4.05	289 ± 4.08	0.23
pH	0.06	0.35	8.87 ± 0.14	8.68 ± 0.13	0.32
ORP (mV)	0.28	0.57	86.8 ± 20.5	40.1 ± 10.1	0.07
NH_4_^+^ (mg/L)	0.02	0.22	0.14 ± 0.08	0.21 ± 0.08	0.55
Cl^-^ (mg/L)	0.99	0.99	20.8 ± 0.25	4.1 ± 0.25	< 0.001

For each model, the marginal R^2^ (i.e., variance explained by the fixed effect of treatment) and Conditional R^2^ (i.e., variance explained by the full model fixed and random effects) are presented, and statistical differences between lakes, determined by the estimated marginal means method, are presented as *p* - values.

### 3.2 (Q2) dominant plant type observational study

**Figure 2:**
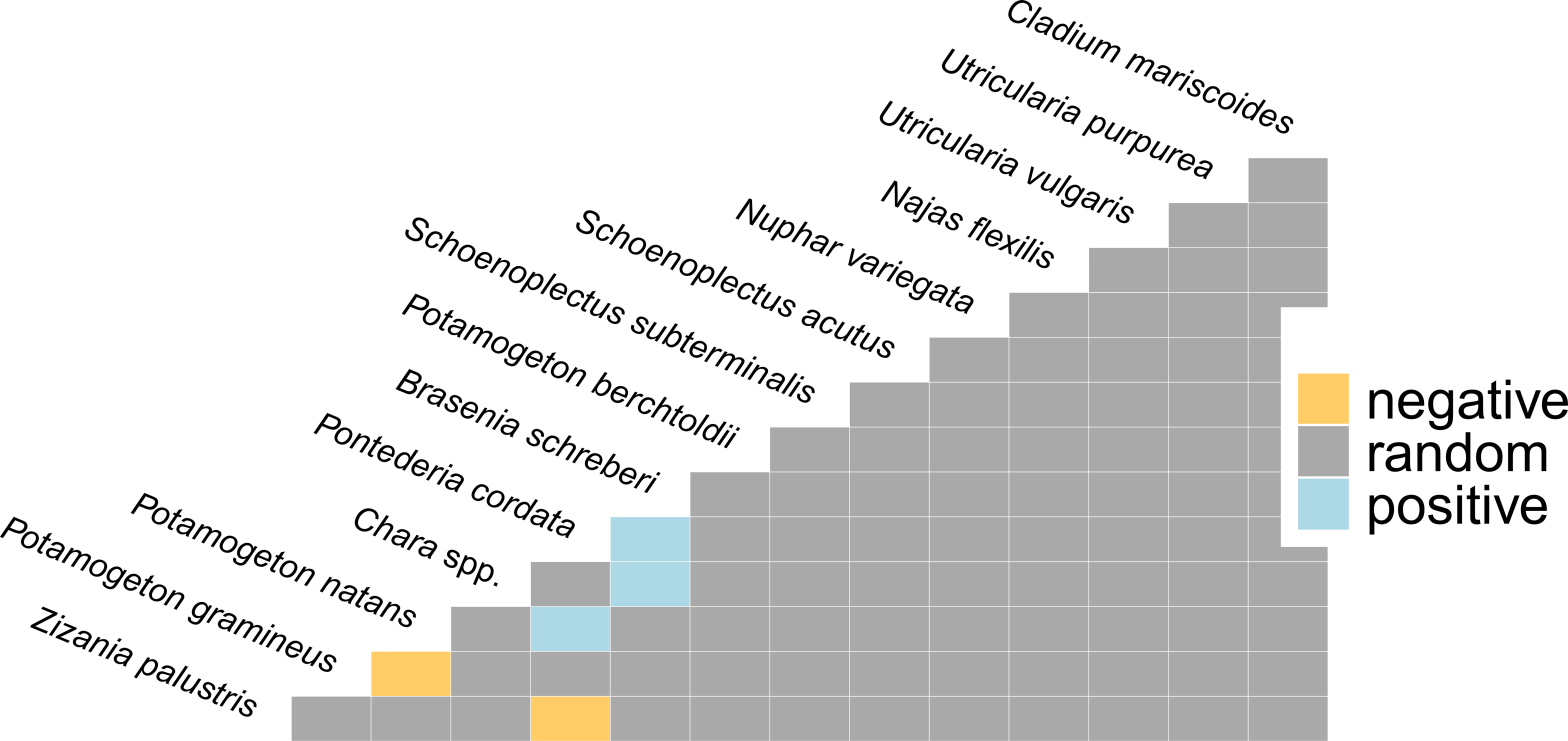
Species co-occurrence matrix of the existing plant and algal species observed in French Farm Lake in 2021-2022 (prior to experimental seeding). Cell color indicates negative, positive, or no association (*p* < 0.05) between each species as determined using species co-occurrence analysis.

**Figure 3:**
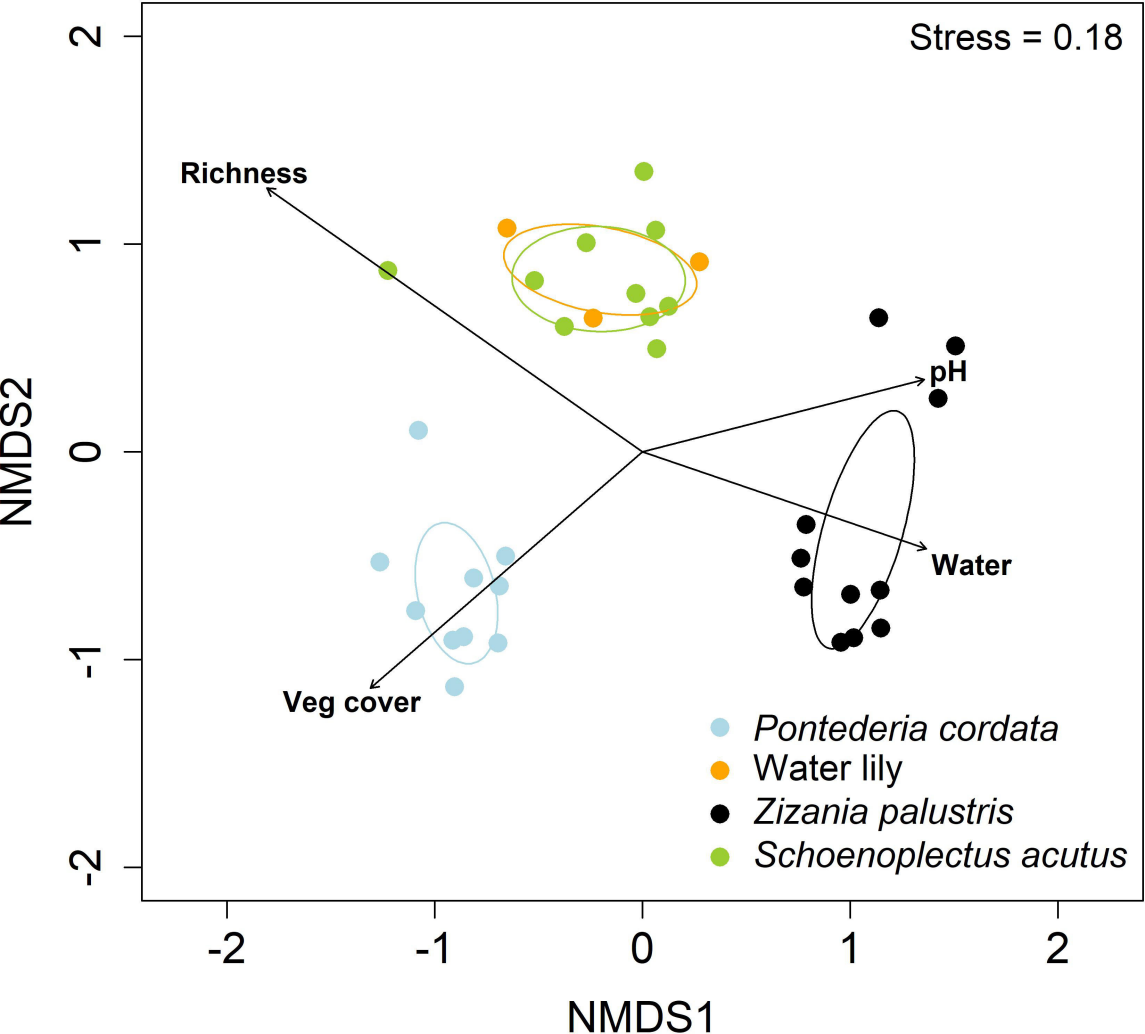
Non-metric multidimensional scaling ordination of 2021–2022 French Farm Lake vegetation sampling (Q2: plant community observational study). The ellipses represent a 95% confidence interval around the mean plot locations of plant community types (water lily [*Nuphar variegata* and *Nymphaea odorata*], *Schoenoplectus acutus, Pontederia cordata*, and *Zizania palustris* [manoomin]) in ordination space. The fitted vector arrows (species richness, water pH, water depth, and total vegetation cover) are significant (*p* < 0.05) and their length is proportional to explanatory strength.

None of the measured environmental variables (water depth, water temperature, pH, ORP) differed by plant community type (all *p* > 0.05; **Table 2**). Manoomin percent coverage and manoomin stem count were significantly higher in manoomin plots compared to *P. cordata* plots (*p* < 0.01; *p* < 0.001) or *S. acutus* plots (*p* < 0.01; *p* < 0.001); no significant differences were found between water lily and the other three community types for either metric. Species richness was significantly lower in manoomin plots compared to the other plant community types (*N. variegata*, *p* = 0.02; *P. cordata*, *p* = 0.01; *S. acutus*, *p* < 0.001); manoomin plots only exhibited 67% of the richness in *P. cordata* plots, 58% of the richness in *S. acutus* plots, and 52% of the richness in water lily plots. Shannon diversity was significantly affected by plant community type at French Farm Lake (*p* = 0.03), with *P. cordata* trending toward lower diversity than *S. acutus* plots (*p* = 0.05). The random factor, year, explained a negligible amount of the variance in the LME models for Shannon Diversity, richness, or manoomin percent coverage (i.e., marginal and conditional R^2^ values did not differ; **Table 2**). However, for manoomin stem count, 23.3% of the variance in the model was explained by the random effect year, and 67.8% by both year and the fixed effect of plant community type (**Table 2**, **Figure 2**). Additionally, species co-occurrence analysis (**Figure 2**) indicated that manoomin presence was negatively correlated with *P. cordata* presence. Non-metric multidimensional scaling ordination (NMDS; **Figure 3**) with a stress value of 0.18 indicated a clear separation of the *P. cordata* and manoomin communities from the other communities in ordination space, whereas the *S. acutus* and water lily communities overlapped almost entirely. The NMDS also demonstrated that several environmental factors varied significantly across ordination space: water depth was positively correlated with manoomin plots (*p* = 0.02); total vegetation cover was positively correlated with *P. cordata* plots (*p* < 0.01); the species richness vector was orthogonal to the total vegetation cover vector (*p* < 0.001); and pH varied with NMDS axis 1.

**Table 2:** Response variables with Marginal R^2^, Conditional R^2^, estimated marginal mean, and standard error by four different plant community types.

Variable	Model fit (R^2^)		Treatment (estimated marginal mean ± SE)			
	Marginal	Conditional	Water lily	*Pontederia cordata*	*Schoenoplectus acutus*	Manoomin
Manoomin cover (%)	0.560	0.560	0.73 ± 1.18^ab^	0.26 ± 0.37^a^	0.32 ± 0.36^a^	5.62 ± 1.46^b^
Manoomin stems (#/m^2^)	0.581	0.678	4.32 ± 3.83^ab^	0.36 ± 0.78^a^	1.08 ± 1.32^a^	13.93 ± 4.63^b^
Richness	0.464	0.464	4.58 ± 0.82^a^	3.58 ± 0.30^a^	4.11 ± 0.34^a^	2.40 ± 0.19^b^
Shannon Diversity	0.234	0.234	0.82 ± 0.27^a^	0.39 ± 0.09^a^	0.80 ± 0.12^a^	0.49 ± 0.09^a^
Environmental metrics						
Water temperature (°C)	0.002	0.836	23.9 ± 0.3	25.4 ± 0.7	25.4 ± 0.5	25.4 ± 0.6
Water depth (cm)	0.088	0.256	59.0 ± 5.2	52.7 ± 5.0	61.0 ± 6.4	67.6 ± 5.9
pH	0.169	0.169	8.3 ± 0.1	8.1 ± 0.1	8.3 ± 0.1	8.4 ± 0.1
ORP (mV)	0.024	0.553	-33.9 ± 24.4	33.7 ± 22.3	34.9 ± 15.0	39.6 ± 17.6

Non-overlapping superscript letters between treatment types indicate statistically significant differences.

### 3.3 (Q3) dominant plant type experimental seeding study

We found significant variability in measured manoomin responses (manoomin density, manoomin cover, and manoomin height; all *p* < 0.001) one-year post seeding in different plant communities at French Farm Lake (**Table 3**). Specifically, seeding in open water areas (outside of wetland plant beds), and in *P. cordata* stands resulted in lower total manoomin stem count, cover, and stem height than in *N. variegata* communities (all *p* < 0.01). Seeding in *S. acutus* stands resulted in intermediate values with greater total stem counts than open water (*p* < 0.01), no difference in total cover from the other community types, and lower average stem height than *N. variegata* plots (*p* = 0.03; **Table 3**). Manoomin associated with *N. variegata* communities were, on average, 19-times, 21-times, and 196-times greater in height than those found associated with *P. cordata*, *S. acutus*, and open water, respectively. Water depth was greater in the open water than in the *P. cordata* and the *S. acutus* community types (both *p* < 0.05) but did not differ among the three plant communities sampled (**Table 3**).

**Table 3:** Manoomin responses one-year following experimental field seeding study with Marginal R^2^, Conditional R^2^, estimated marginal mean, and standard error by four community types (i.e., treatment).

Variable	Model fit (R^2^)		Treatment (estimated marginal mean ± SE)			
Manoomin metrics	Marginal	Conditional	Open water	*Nuphar variegata*	*Pontederia cordata*	*Schoenoplectus acutus*
Density (stems/m^2^)	0.16	0.71	0.01 ± 0.07^a^	0.87 ± 0.50^c^	0.10 ± 0.67^ab^	0.27 ± 0.58^bc^
Cover (%)	0.33	0.41	0.03 ± 0.06^a^	0.71 ± 0.35^b^	0.10 ± 0.59^a^	0.29 ± 1.02^ab^
Height (cm)	0.12	0.50	0.15 ± 0.30^a^	7.23 ± 8.13^b^	0.31 ± 0.48^a^	0.27 ± 0.44^a^
Environmental metrics						
Water depth (cm)	0.16	0.26	62.8 ± 10.1^a^	53.5 ± 3.6^ab^	38.6 ± 1.8^b^	40.3 ± 5.0^b^

Non-overlapping superscript letters between plant community types indicate statistically significant differences.

### 3.4 (Q4) litter and shading mesocosm experiment

**Figure 4:**
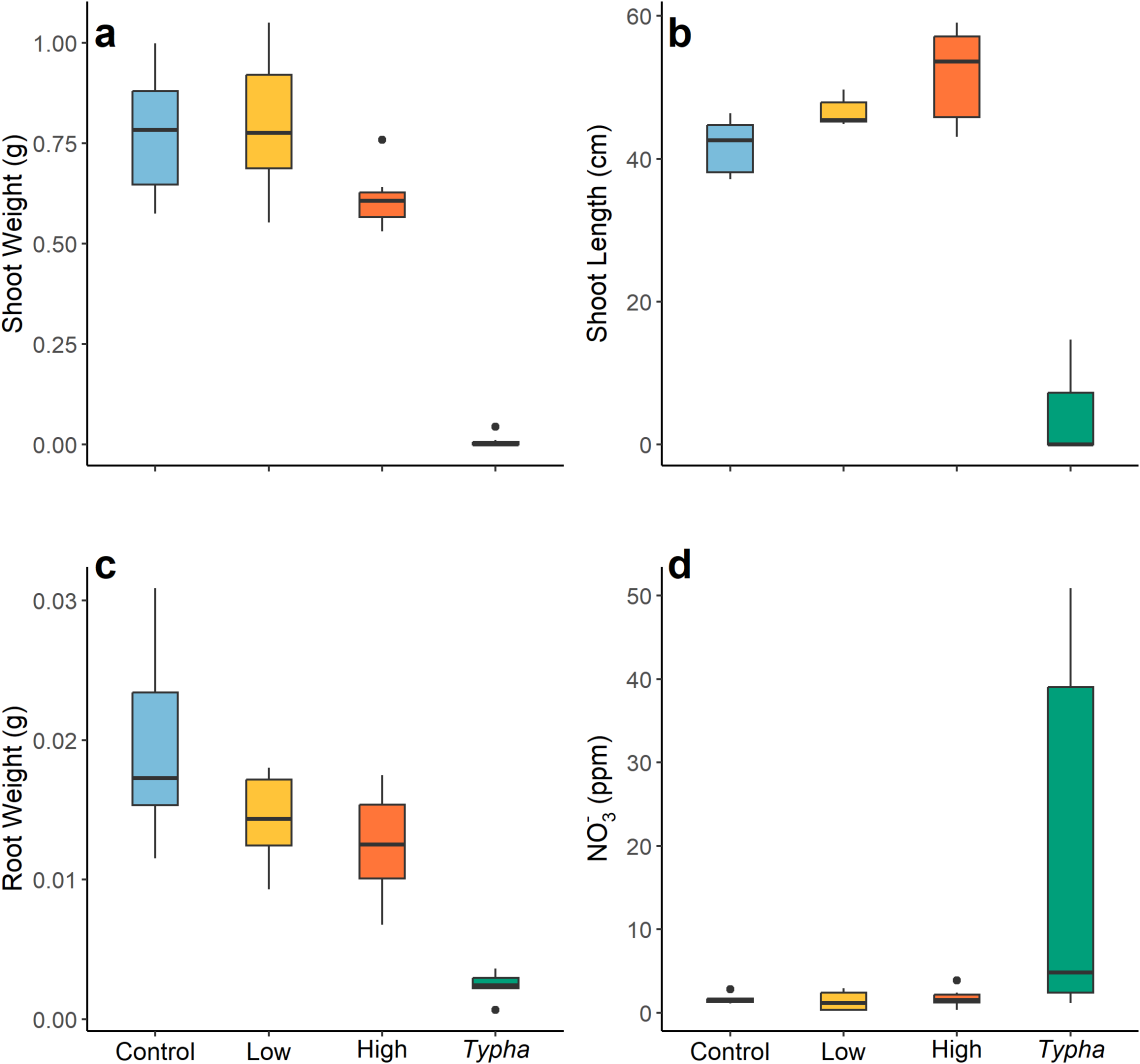
Response variables of mesocosm experiment by treatment level (control, low cover [Low; 40% shading], high cover [High; 80% shading], and *Typha* litter [*Typha*]): manoomin shoot weight **(a)**, manoomin shoot length **(b)**, manoomin root weight **(c)**, and water NO_3_^-^ concentration **(d)**.

The mesocosm study indicated that the *Typha* litter treatment had a significant effect on all reported metrics of manoomin growth and water nutrient concentrations (*p* < 0.001; **Table 4**, **Figure 4**). Across several response variables, *Typha* treatment resulted in greater differences in manoomin response variables than shading treatments. For example, manoomin root weights in control treatments were approximately 1.4-times greater than in low cover, 1.7-times greater than in high cover, and 10.0-times greater than in the *Typha* treatment level. Manoomin shoot weights in control, low cover, and high cover treatments were significantly higher than *Typha* litter treatments (all *p* < 0.001). Manoomin shoots in the high cover treatment were also significantly lower in weight than those grown in the low cover treatment (*p* = 0.03). Manoomin root: shoot ratio was 4.9-times higher in the *Typha* treatment compared to the control. Water NO_3_^-^ concentration was also significantly higher in *Typha* treatment buckets than in all other treatments (all *p* < 0.05).

**Table 4:** Response variables with Marginal R^2^, Conditional R^2^, estimated marginal mean, standard error, and *p*-values, by four different treatments levels (control, low cover (40%), high cover (80%), and *Typha × glauca* litter).

Variable	Model fit (R^2^)		Treatment (estimated marginal mean ± SE)			
	Marginal	Conditional	Control	Low Cover	High Cover	*Typha × glauca*
Manoomin metrics						
Total weight (g)	0. 944	0. 960	1.204 ± 0.081^a^	1.138 ± 0.079^a^	0.888 ± 0.070^b^	0.018 ± 0.010^c^
Shoot weight (g)	0. 949	0. 970	0.766 ± 0.054^ab^	0.791 ± 0.055^a^	0.610 ± 0.048^b^	0.003 ± 0.033^c^
Root weight (g)	0.796	0.853	0.020 ± 0.002^a^	0.014 ± 0.002^ab^	0.012 ± 0.001^b^	0.002 ± 0.001^c^
Root: Shoot	0.746	0.786	0.558 ± 0.096^a^	0.461 ± 0.079^a^	0.338 ± 0.058^a^	2.720 ± 0.762^b^
Shoot length (cm)	0.937	0.964	41.7 ± 1.9^a^	46.6 ± 1.9^ab^	51.7 ± 1.9^b^	4.2 ± 1.9^c^
Shoot count	0. 588	0.588	22.91 ± 2.13^a^	24.09 ± 2.19^a^	21.89 ± 2.08^a^	1.05 ± 0.46^b^
Spindliness (stem height: stem mass)	0.860	0.872	54.8 ± 7.36^a^	59.5 ± 8.00^a^	84.3 ± 11.34^a^	840.5 ± 180.97^b^
Environmental metrics						
Water NO_3_^-^ (ppm)	0.391	0.550	1.40 ± 0.60^a^	0.98 ± 0.39^a^	1.47 ± 0.59^a^	8.15 ± 3.27^b^

Non-overlapping superscript letters between treatment types indicate statistically significant differences.

## 4 Discussion

Variable environmental conditions within and between lakes and competitive exclusion of manoomin are persistent factors limiting productivity and the local success of seeding efforts (Lee, 1986). Here, we attempted to shed light on lake-to-lake differences, and invasive and native species competitive effects on manoomin by synthesizing four recent studies. We compared the environmental conditions and plant growth between two lakes with disparate manoomin seeding success (Q1). Additionally, we sought to determine the effects of plant competition from a suite of native plants on the germination and productivity of manoomin in a northern Michigan lake (Q2, Q3). Finally, we evaluated the indirect effects of shading and litter from an invasive plant on manoomin germination and early growth through a controlled mesocosm experiment (Q4). We found that the lake with higher water temperatures (Wycamp Lake) also had lower measures of all manoomin variables. Additionally, we found that both native species (*Pontederia cordata*) and non-native species (*Typha*) limited manoomin germination and productivity.

### 4.1 Lake conditions influence manoomin (Q1)

As expected from field observations, we found that manoomin stands within French Farm Lake were more robust, in terms of stem count, mass, and height, than within Wycamp Lake. The higher degree of spindliness observed in manoomin at Wycamp Lake (greater than at French Farm Lake by a factor of 2.5) also suggested conditions within Wycamp were stressful to manoomin (Atkins, 1986; Varty et al., 2024). Varty et al. (2024) reported a 2.5 °C greater spring (May) water temperature in Wycamp than in French Farm Lake. Similarly, we found a significant difference in summer water temperature between lakes, on average 1.7 °C greater in Wycamp Lake than in French Farm Lake. Warmer lake temperatures likely contributed to the divergent outcomes of manoomin populations. Longer lake-ice duration, which would be expected in cooler lakes, has been linked with greater manoomin stem density the following season (Nyblade et al., 2025). Further, higher water temperatures are associated with decreased manoomin germination and growth (Varty et al., 2024). In addition, while we found that Cl^-^ concentrations were significantly greater in French Farm Lake, levels in both lakes were well below those documented to negatively impact manoomin (Fort et al., 2014; Varty et al., 2024) indicating that differential growth was not likely a result of lake Cl^-^. A more extensive regional study of seasonal water temperature dynamics from lakes with variable manoomin productivity is necessary to draw broader conclusions about the role of water temperatures on manoomin across its range. Further, additional controlled studies precisely manipulating water temperature would provide insights into manoomin temperature tolerances and thresholds.

### 4.2 Extant native plant stands impact manoomin establishment and occurrence

Our two-year observational study at French Farm Lake (Q2) supported our hypotheses that in a lake with a self-sustaining manoomin population, manoomin density and productivity varied depending upon the dominant plant species with which it co-occurred. We found no statistical differences in measured environmental variables among plant communities, providing further support for our attribution of plant community type as a driving mechanism for differences in manoomin. Plant richness was lower in manoomin dominated plots than in other plant stands, reflecting manoomin’s propensity to thrive with less interspecies competition (Atkins, 1986; Lee, 1986). In particular, while manoomin was intermixed within all dominant plant types sampled, we found substantially lower cover and density in native plant stands (water lily, *P. cordata*, and *S. acutus*) than in manoomin stands, and a significant negative co-occurrence with *P. cordata*. In our experimental seeding study (Q3), we found lower manoomin density and biomass in *P. cordata* than in *N. variegata* (yellow pond-lily) stands and seeded manoomin grew taller in *N. variegata* stands than in *P. cordata* or *S. acutus* stands. The negative relationship between multiple measures of manoomin growth and *P. cordata* from both studies indicates competitive exclusion as a likely mechanism, whereby manoomin has a reduced capacity for reproductive success in the presence of *P. cordata*. Additional controlled experiments are necessary to determine the precise drivers of manoomin competitive exclusion. In contrast, we found that co-occurrence with *N. variegata* is somewhat less detrimental to manoomin growth and reproduction. These findings reflect those of Heubler and Gedan (2024), who found that while *N. advena* competed with *Z. aquatica* for light and reduced its biomass, *N. advena* also provided associational resistance from *Branta canadensis* (Canada geese) grazing pressure. The random effect of year explained a negligible amount of the variation in most measures across the two-year observational study. The exception to this pattern was manoomin stem count, in which year explained 23% of variability, consistent with observed patterns of year-to-year oscillations in manoomin productivity (Walker et al., 2010). In order to extrapolate our findings to explain long-term manomin dynamics, annual monitoring of manoomin populations among stands of mixed aquatic vegetation is necessary.

### 4.3 Invasive plant and shade impacts on manoomin

In our mesocosm study, we found that manoomin germination and early growth was negatively impacted by *Typha* litter and a proxy for high levels of floating plant shading, supporting our hypotheses and identifying shading as a partial mechanism by which native and non-native plants outcompete manoomin. The *Typha* litter treatment had the strongest impacts, significantly reducing manoomin above and belowground biomass, stem and root length, and shoot count, and increasing root: shoot ratio and plant spindliness compared to controls. Further, in 4 of the 7 *Typha* replicates, germinated manoomin seeds failed to develop shoots, suggesting the *Typha* litter created an environment highly unfavorable to manoomin growth, though the precise causal mechanism for this effect was not determined. Interestingly, water NO_3_^-^ concentrations were over 5-times greater in the *Typha* litter treatments than in controls. *Typha* litter can be an important component of internal nitrogen loading in wetland environments (Farrer and Goldberg, 2009; Larkin et al., 2012b). The decomposition of *Typha* litter introduces mineralized plant-available nitrogen in the environment, in the forms of NH_4_^+^ and NO_3_^-^, which in turn can create a positive feedback loop, in which high-nutrient conditions continue to favor growth of *Typha* over other wetland plants due to *Typha*’s elevated uptake efficiency and productivity (Larkin et al., 2012b). As an annual plant manoomin relies on sediment and, to a limited extent, water column nutrients for growth and manoomin productivity and seed production is limited by availability of N (Sims et al., 2012). Therefore, the additional water column N in *Typha* treatments was not likely the direct causal factor for reduced manoomin growth. Rather, the observed NO_3_^-^ spike was more likely a result of *Typha* litter decomposition (Farrer and Goldberg, 2009).

Shade treatments had less of an impact on manoomin than *Typha* litter. No manoomin measures differed significantly between controls and the low cover treatment (40% cover). However, the high cover treatment (80% cover) caused a reduction in total average manoomin biomass and root biomass and an increase in shoot length compared to controls. Indeed, shading is a primary mechanism by which some invasive floating plants, like European frogbit, (*Hydrocharis morsus-ranae*; EFB) negatively impacts native plants (Catling et al., 2003). Further research into the effects of shading and EFB, in particular, on manoomin is warranted, given that EFB continues to spread throughout the Great Lakes region (Zhu et al., 2018; Monks et al., 2019).

### 4.4 Future studies

Our studies helped to identify several areas for future research. In particular, future lake-to-lake comparison studies would benefit from a paired seeding design and multiple lakes, wherein seeds from a common source are sown into multiple lakes and tracked through the following growing season. Second, additional controlled plant-to-plant competition studies that include more detailed water chemical analyses are necessary to isolate the causal factors for apparent impacts of *Typha* litter and shading, and negative co-occurrences between manoomin and *Pontederia cordata*. Finally, while our mesocosm results were clear, additional field experiments are necessary to test the effects of litter and shading on manoomin in natural water bodies.

### 4.5 Management implications

Our studies indicate that multiple factors associated with global change (specifically warming lake water temperatures and non-native plant encroachment) as well as native plant competition can be detrimental to the germination and growth of manoomin. In particular, care should be taken to avoid seeding manoomin in and around *P. cordata* and invasive *Typha* stands. *P. cordata* negatively co-occurred with manoomin and invasive *Typha* and its litter caused particularly strong negative effects on manoomin germination and early growth. Thus, management of invasive *Typha* and the removal of its abundant litter are necessary to allow for the establishment of new manoomin stands and for the sustainable management of existing manoomin populations. Cutting invasive *Typha* below water and mechanically harvesting its litter are particularly effective control techniques (Lishawa et al., 2017, Lishawa et al., 2023), which could be coupled with manoomin restoration efforts.

Our findings illustrate the complexity involved in restoring a native annual species to a natural freshwater ecosystem with extant native plant populations. Further, our study highlights the multifaceted threats that global change places on native plant species of high cultural importance. Indigenous peoples have stewarded manoomin populations in the Great Lakes region for millennia. We hope that the particular factors affecting manoomin restoration that our study has identified will be incorporated into the significant knowledge already held by these communities, to the benefit of manoomin, people, and the aquatic ecosystems of the region.

## Data Availability

Data are publicly available in the Dryad Data Depository: https://doi.org/10.5061/dryad.05qfttfj6.
